# Inhibition of vacuum sublimation artefacts for (Scanning) Transmission Electron Microscopy ((S)TEM) of sulphur samples *via* encapsulation

**DOI:** 10.12688/openreseurope.14378.1

**Published:** 2022-01-04

**Authors:** Oskar Ronan, Clive Downing, Valeria Nicolosi

**Affiliations:** 1 Advanced Materials and Bioengineering Research (AMBER), Trinity College Dublin, Dublin, Ireland; 2 Centre for Research on Adaptive Nanostructures and Nanodevices (CRANN), Trinity College Dublin, Dublin, Ireland; 3 School of Chemistry, Trinity College Dublin, Dublin, Ireland; 4 Advanced Microscopy Laboratory, Trinity College Dublin, Dublin, Ireland

**Keywords:** Transmission Electron Microscopy (TEM), sulfur, in-situ, sublimation, gas cell.

## Abstract

Lithium-sulfur battery is one of promising candidates for next-generation energy storage device due to the sulfur cathode material with low cost and nontoxicity, and super high theoretical energy density (nearly 2600Wh kg ^−1^) and specific energy (2567Wh kg ^−1^).

Sulphur, however, poses a few interesting challenges before it can gain widespread utilisation. The biggest issue is known as the polysulphide shuttling effect which contributes to rapid capacity loss after cycling. Accurate characterisation of sulphur cathodic materials becomes critical to our understanding polysulphide shuttling effect in the quest of finding mitigating solutions. Electron microscopy is playing a crucial role in battery research in determining structure–property–function relations. However, sulphur undergoes sublimation at a point above the typical pressures found in the column of a transmission electron microscope (TEM) at room temperature. This makes the imaging and characterisation of any sort of nanostructured sulphur samples challenging, as the material will be modified or even disappear rapidly as soon as it is inserted into the TEM vacuum. As a result, materials characterised by such methods are prone to deviation from normal conditions to a great extent. To prevent this, a novel method of encapsulating sulphur particles between silicon nitride (SiN _x_) membranes is demonstrated in this work.

## Introduction

With increased interest in sulphur as a candidate cathode material for high-performance lithium-ion batteries, the characterisation of sulphur to determine structure–property–function relations has become increasingly important in recent years ^1– 3^. The characterisation of sulphur by electron microscopy poses a number of interesting challenges. The foremost issue is the high-vacuum environment required for electron microscopy; in the low pressure regime sulphur may undergo sublimation under high vacuum at room temperature, rendering it challenging to image in the transmission electron microscope (TEM) ^4– 6^.

In order to prevent sublimation and give the microscopist enough time to complete imaging before the sample disappears entirely, a sample must be held in the solid region of its phase diagram ^5– 7^. This can be achieved in a number of ways for samples that can undergo sublimation; by cryogenically cooling the sample to a temperature past the sublimation line, by increasing the pressure surrounding the sample to a level greater than its equilibrium vapor pressure (for sulphur; ~10 ^-7^ Torr @ 20°C), or a combination of both ^4, 5, 7^.

At the typical TEM vacuum pressures (approx. 7 × 10 ^−8^ Torr), sulphur exists in the gas phase ^4, 8^. To overcome this issue, cryogenic cooling to below 250K or increasing pressure around the sample is required to keep sulphur in the solid orthorhombic phase ^4, 5, 7^.

Both methods have been previously demonstrated by Levin *et al.* ^5^, showing stable imaging of sulphur using a Cryo-holder for the TEM and using an ambient pressure SEM. Building on this work, a novel method for imaging sulphur in the TEM using a SiN _x_ window gas cell holder is here proposed. With the introduction of commercially available gas cell holders, a sample can be held at a set pressure with any compatible gas connected to the sample holder. In this work, the sample was left open to air at atmospheric pressure. Given that the only necessary modification to the system would be the pressure, a set pressure of any non-reactive gas (such as N _2_ or Ar) would also work. The concept of protectively encapsulating a sample to inhibit degradation in the microscope environment has been used in previous work by Kwon *et al.* to limit oxidation of copper nanowires, and a conceptually similar solution is demonstrated in this work ^9^.

## Methods

The sulphur particles were observed *via* field-emission (scanning) transmission electron microscopy ((S)TEM) (Titan, Thermo Fisher Scientific Inc.). The macrostructure and elemental composition of the sulphur particles were analysed using (S)TEM (Titan, Thermo Fisher Scientific Inc.) coupled with simultaneous energy dispersive X-ray spectroscopy (EDX; Ametek 30mm ^2^ EDX detector). A vacuum of 7 × 10 ^−8^ Torr and an acceleration voltage of 300 keV were employed for both TEM and STEM imaging during the measurements.

A sample of elemental sulphur was prepared by dispersing the as purchased sulphur powder (325 mesh, 99.5% purity, Alpha Aesar) in isopropanol (IPA) *via* ultrasonic bath sonication (Fisherbrand 11207 operated at 37kHz) ^10^. The dispersion was deposited on a lacey carbon 400 mesh Cu grid (01896-F, TED PELLA Inc.) by dropcast for observation in vacuum, and allowed to dry in air. Sulphur particles were deposited onto Climate E-chips (P.T.GH.SS.2, DENS Solutions B.V. Delft, Netherlands, shown in Figure 4) in the same manner. Each chip, which consisted of an ~30-nm-thick SiN _x_ membrane, was mounted in a Climate TEM holder (DENS Solutions B.V. Delft, Netherlands.). Here the SiN _x_ membrane acts as sample support while simultaneously isolating the sample from the vacuum environment.

The sample was loaded into the microscope, and a sulphur particle was chosen as the region of interest. The column valves were closed between each consecutive image to reduce any beam-induced artefacts for both standard Cu grid experiment and SiN _x _window encapsulated experiment.

The encapsulated sample was left open to atmosphere at 1bar during the experiment.

## Results

**Figure 1.**
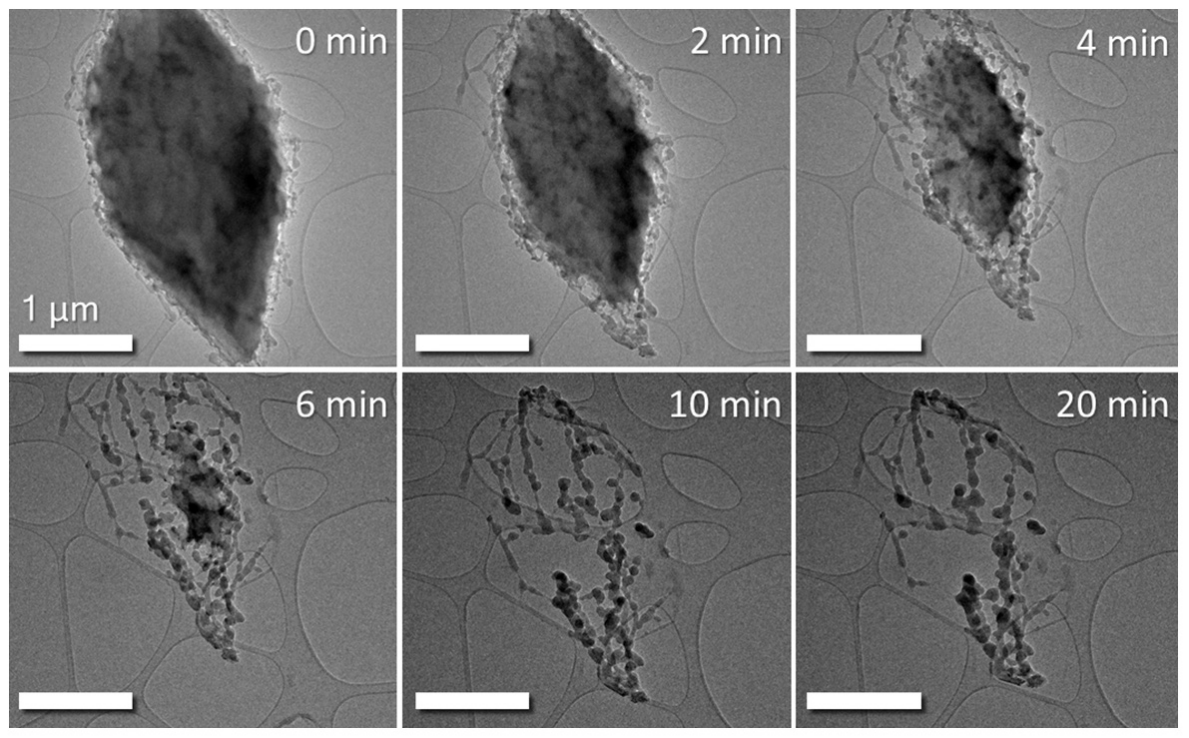
BF TEM time series images of sulphur particle subliming in TEM column vacuum of 7x10 ^-8^ Torr. The large particle in the region of interest is seen to dramatically shrink over time under vacuum exposure. Column valves were closed between images to prevent beam-induced effects altering the sublimation process. Scale bar is 1µm in all above images.

**Figure 2.**
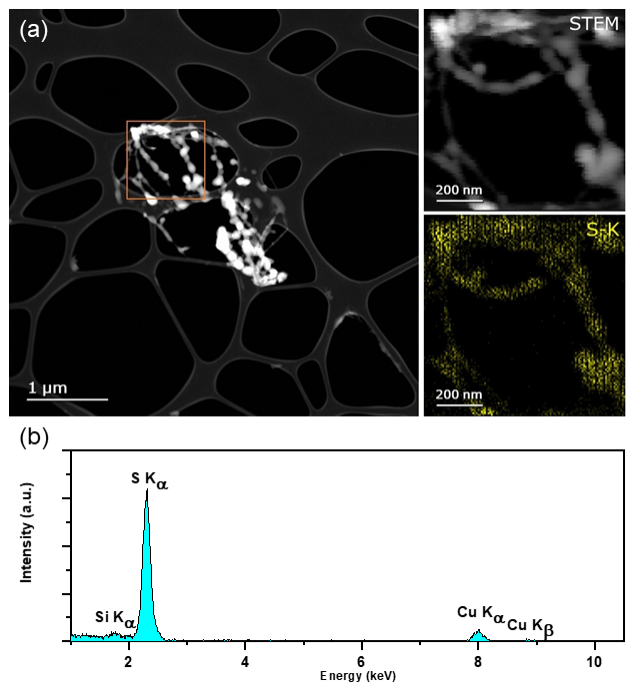
**a**) HAADF STEM of residual S post-sublimation acquired on FEI Titan @300keV. Inset shows STEM image of highlighted ROI and EDX maps of S corresponding to the EDX spectrum. **b**) Integrated spectrum of region of interest, confirming elements listen in a) are present. Maps confirm that the residue is indeed composed of sulphur. (Si peak due to an EDX spectral artefact; an internal fluorescence peak from the silicon window on the silicon drift detector likely. Cu peak is a background signal from the TEM grid.)

In order to demonstrate sublimation of sulphur in normal TEM vacuum conditions a sulphur sample was prepared as described above and introduced into the microscope. As can be seen in the bright-field (BF) TEM images in Figure 1, the sulphur particle undergoes sublimation in the column vacuum over a period of time as little as 10 minutes. After this time, the residual material becomes vacuum-stable and does not sublimate further. It is believed to be super-sublimated polymeric sulphur, according to literature sources ^11, 12^. This is known to have a much lower vapour pressure than elemental sulphur ^11^ and is comparable to the experiments conducted by Levin *et al.* ^5^. Examination of the residue by EDX STEM mapping at 300keV confirms the residue as sulphur as evidenced by the S-K _α _peak at 2.3keV ( Figure 2).

**Figure 3.**
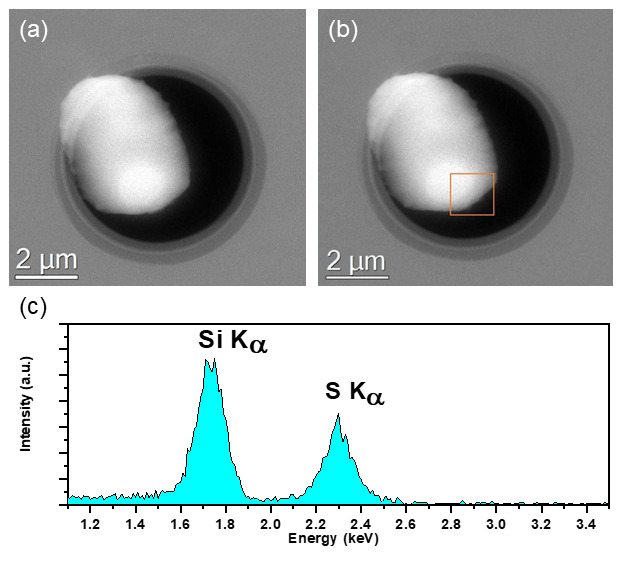
HAADF STEM images of a sulphur particle before ( **a**) and after ( **b**) a 2-hour exposure inside the TEM column vacuum. The particle is isolated from the column vacuum by the SiN _x_ window of the gas cell chips, thus preventing sublimation of the sample. ( **c**) Integrated EDX Spectra of highlighted ROI of sulphur particle on SiN _x_ window of the gas cell chips after 2-hour exposure shown in ( **b**) acquired @300keV in FEI Titan equipped with an Ametek 30mm ^2^ EDX detector. S and Si peaks from the S particle and SiNx window respectively clearly detected.

In order to investigate the potential of the commercial gas cell sample holder MEMS chips ^13, 14^ as a method to image the sulphur without sublimation the sample was deposited on SiN _x_ window DENS Solutions Climate MEMS chips (DENS Solutions B.V. Delft, Netherlands.), inserted into the column, and imaged over a period of 2 hours using the same experimental conditions as the previous experiment (300keV TEM and STEM will a dwell time of 20ms/pixel and beam current of 0.5nA for EDX analysis). Unlike the previous experiment, the initial state of the particle is maintained in the column vacuum, and no change in particle morphology was observed over the observation period of 2 hours (as seen in Figure 3) in the high-angle annular dark field (HAADF) STEM image. The particle was kept at ambient pressure (1bar) inside the Climate holder, and the column valves were closed between image acquisitions to reduce any beam-induced artefacts ^12^.

**Figure 4.**
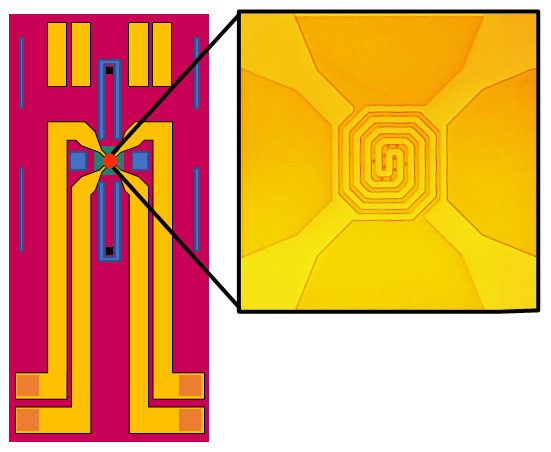
Schematic view of DENS Solutions Climate SiN _x _window chips. SiN _x _windows isolate the gas flow inside the holder from the vacuum of the TEM column. Heating coil visible over SiN _x _windows.

The EDX spectrum acquired after the 2 hours showing S-K _α_ and Si-K _α_ peaks (located at 2.3keV and 1.74keV respectively) in Figure 3c confirms that the particle is indeed sulphur supported on a SiN _x_ window. The suppression of the sublimation clearly demonstrates the viability of the use of windowed in-situ sample holders for imaging and characterisation of high vapour pressure materials in the TEM.

Being an electrical insulating material, sulphur is still very susceptible to radiolytic ionisation damage from the electron beam, and careful consideration must be made to the beam current passing through the sample for both quantitative and qualitative spectroscopy ^15 –18^. For example, in order to acquire data for an EDX map of the sulphur particle, higher beam doses (using longer pixel dwell times or a higher beam current) must be used in order to generate enough counts for the detector. This has the effect of damaging the sample heavily due to sulphur’s susceptibility to knock-on damage, and alternate methods must be employed to characterise the elemental composition of the sample with this method ^15– 18^. It is still possible to collect spectra to confirm elemental composition of the sample *via* EDX using a lower dose ^19, 20^, as can be seen in Figure 3. In this regard, the cryo-EM method utilised by Levin *et. al.* ^5^ has its benefits over the SiN _x_ window approach that can be found in many in-situ holders. Cryogenic electron microscopy has the secondary benefit of slowing or even inhibiting beam induced artefacts and sublimation artefacts simultaneously, allowing for easier collection of mapping data ^21^.

## Conclusions

The ability to image potentially vacuum sensitive materials for energy storage applications such as sulphur composites without sublimation artefacts creates new possibilities for their characterisation. To control sublimation in TEM analysis, an encapsulation method utilizing a commercial gas cell TEM sample holder is demonstrated. Where before, samples would sublime before imaging could take place, this novel encapsulation method allows for sample stability over the time frames necessary for both imaging, and spectroscopic analysis by EDX, or even EELS with ultra-thin SiN _x_ windows. The proposed method is suitable for the TEM analysis of other specimens that may undergo sublimation at TEM vacuum pressures, such as zinc, magnesium, or some polymers to name a few. Such techniques would have been impossible for such materials in the past and presents exciting new opportunities for battery materials researchers.

## Data availability

### Underlying data

Open Science Framework: Inhibition of vacuum sublimation artefacts for (Scanning) Transmission Electron Microscopy ((S)TEM) of sulphur samples via encapsulation, https://doi.org/10.17605/OSF.IO/TRF3B.

This project contains the following underlying data:

- Fig 1_1.jpg
- Fig 1_2.jpg
- Fig 1_3.jpg
- Fig 1_4.jpg
- Fig 1_5.jpg
- Fig 1_6.jpg
- Fig 2_1.tif
- Fig 2_2.tif
- Fig 2_3.tif
- Fig 2_Integrated EDX spectrum.csv
- Fig 3.tif
- Fig 3_1.tif
- Fig 3_Integrated EDX spectrum.csv

Data are available under the terms of the Creative Commons Zero "No rights reserved" data waiver (CC-By 4.0 Public domain dedication).
