# Supplementary material for: Inhibition of vacuum sublimation artefacts for (Scanning) Transmission Electron Microscopy ((S)TEM) of sulphur samples via encapsulation
Source: Open Res Eur. 2022 Feb 1;2:1. Originally published 2022 Jan 4. [Version 2] doi: 10.12688/openreseurope.14378.2 (PMC10445806; doi:10.12688/openreseurope.14378.2)
Supplement: Peer review history

# Peer review: Reviewer response for version 2

**DOI:** 10.21956/openreseurope.15606.r28481

**Published:** 2022-02-17

**Authors:** Toma Susi

**Affiliations:** 1 Faculty of Physics, University of Vienna, Vienna, Austria

Thanks to the authors for the revisions – the new discussion could have been a little more extensive, but I understand this is not the main point here, and it's fine that the issues are only briefly mentioned.

Is the rationale for developing the new method (or application) clearly explained?

Yes

Is the description of the method technically sound?

Yes

Are the conclusions about the method and its performance adequately supported by the findings presented in the article?

Yes

If any results are presented, are all the source data underlying the results available to ensure full reproducibility?

Partly

Are sufficient details provided to allow replication of the method development and its use by others?

Yes

Reviewer Expertise:

NA

I confirm that I have read this submission and believe that I have an appropriate level of expertise to confirm that it is of an acceptable scientific standard.

# Peer review: Reviewer response for version 2

**DOI:** 10.21956/openreseurope.15606.r28480

**Published:** 2022-02-16

**Authors:** Andrew A. Stewart

**Affiliations:** 1 Department of Physics and the Bernal Institute, University of Limerick, Limerick, Ireland

The article can in my view be accepted, the points have all been addressed and all the data now being available fits into the open ethos of the journal.

Is the rationale for developing the new method (or application) clearly explained?

Yes

Is the description of the method technically sound?

Yes

Are the conclusions about the method and its performance adequately supported by the findings presented in the article?

Yes

If any results are presented, are all the source data underlying the results available to ensure full reproducibility?

Yes

Are sufficient details provided to allow replication of the method development and its use by others?

Yes

Reviewer Expertise:

Transmission Electron Microscopy

I confirm that I have read this submission and believe that I have an appropriate level of expertise to confirm that it is of an acceptable scientific standard.

# Peer review: Reviewer response for version 1

**DOI:** 10.21956/openreseurope.15517.r28318

**Published:** 2022-01-21

**Authors:** Toma Susi

**Affiliations:** 1 Faculty of Physics, University of Vienna, Vienna, Austria

Although I am not an expert on battery materials, the authors convincingly argue for the importance of studying sulphur-based composites. For this purpose, (scanning) transmission electron microscopy and spectroscopy are indeed ideally suited, and the prevention of sulphur sublimation is an experimental challenge that the presented method appears to nicely address.

 The article can in my view be already accepted, although I have some suggestions for improvement that the authors should consider.

 Most importantly, the work would greatly benefit from a balanced discussion of the potential downsides that encapsulation within SiN _x_ membranes might bring. For example, how much more laborious is the sample preparation, and is the area that can be studied limited in an important way? How does the presence of the membrane materials affect imaging resolution or contrast? Does the presence of Si and N in the membranes interfere with EDX or EELS characterisation of some elements that might be important to identify in battery composites?

 As regards to open data, I would urge the authors to not use a lossy compressed file format such as .jpg for any data items. Instead, uncompressed .tif or .png formats should be used, or even better yet, the original data files should also be included without any overlaid graphics.

 Finally, I have minor suggestions for copyediting corrections:

- Use of the acronym in the title seems unnecessary.
- The phrase "super high" in the abstract seems a little unscientific, but perhaps this is common usage in the field.
- Often a space is missing between numerical quantities and units.
- There is some apparent repetition in the text, e.g. the Titan instrument provider is repeated, and the closing of column valves is mentioned twice on p. 3.
- A few typos are present, e.g. in "300keV TEM and STEM **will** a dwell time" on p.3, "confirming elements **listen** in a)" in the caption of Figure 2 on p.4, and "Being an electrical insulating material" (as opposed to "electrical insulator" or "electrically insulat **ing** material") on p.6.

Is the rationale for developing the new method (or application) clearly explained?

Yes

Is the description of the method technically sound?

Yes

Are the conclusions about the method and its performance adequately supported by the findings presented in the article?

Yes

If any results are presented, are all the source data underlying the results available to ensure full reproducibility?

Partly

Are sufficient details provided to allow replication of the method development and its use by others?

Yes

Reviewer Expertise:

Transmission electron microscopy

I confirm that I have read this submission and believe that I have an appropriate level of expertise to confirm that it is of an acceptable scientific standard.

# Peer review: Reviewer response for version 1

**DOI:** 10.21956/openreseurope.15517.r28271

**Published:** 2022-01-18

**Authors:** Andrew A. Stewart

**Affiliations:** 1 Department of Physics and the Bernal Institute, University of Limerick, Limerick, Ireland

This article addresses the important problem of materials with high vapour or sublimation pressures within the low pressure environment of the Transmission Electron Microscope (TEM), suggesting the use of gas cells to encapsulate the material and observe it at atmospheric pressure to circumvent the high vacuum problems caused by the instrumentation. Sulphur is used for the purposes of demonstrating the effectiveness of the gas cell.

 The comparison of the sulphur particles is made with a particles on a carbon substrate and compares the results with the closed cell made of silicon nitride. 

 The figures 1&2 clearly show the disintegration of the particle in the presence of the high vacuum and the electron beam, with figure 3 showing the stability of the particle in the presence of the gas phase at atmospheric pressure. What is omitted is the difficulties of producing such samples where the placement of the sample on the ultra thin windows of the SiN_x chips is either luck or careful placement with a FIB, compared to a carbon grid where almost any region of the grid is reasonable to collect data from. 

 A point not addressed in the text is using the high pressure room temperature approach can also avoid any phase changes as a result of temperature changes. Just using atmosphere can be problematic for some samples which are more sensitive to water vapour and its radicals caused by the electron beam.

 While the data is provided the nature of the data output from some of the vendors software will be missing significant metadata required to fully intemperate the images without further information, this is a general problem of TEM experiments currently. The authors could indicate which software can be used to read the files to obtain the metadata such as Velox or Hyperspy, would be a way of partially addressing this issue.

Is the rationale for developing the new method (or application) clearly explained?

Yes

Is the description of the method technically sound?

Yes

Are the conclusions about the method and its performance adequately supported by the findings presented in the article?

Yes

If any results are presented, are all the source data underlying the results available to ensure full reproducibility?

Yes

Are sufficient details provided to allow replication of the method development and its use by others?

Yes

Reviewer Expertise:

Transmission Electron Microscopy

I confirm that I have read this submission and believe that I have an appropriate level of expertise to confirm that it is of an acceptable scientific standard.
